# Supplementary figures and images for: A simulation-based framework for modeling and prediction of personalized blood pressure trajectories in hypertensive patients after antihypertensive treatment
Source: PLoS One. 2025 Apr 10;20(4):e0318549. doi: 10.1371/journal.pone.0318549 (PMC11984981; doi:10.1371/journal.pone.0318549)

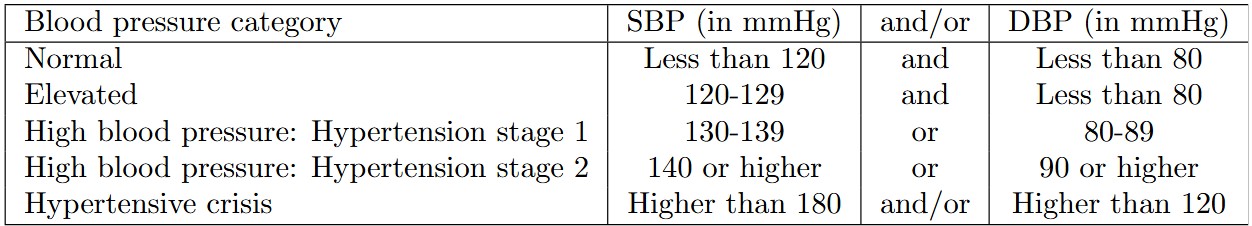

Supplement: S1 Table [file pcbi.0318549.s001.jpg]

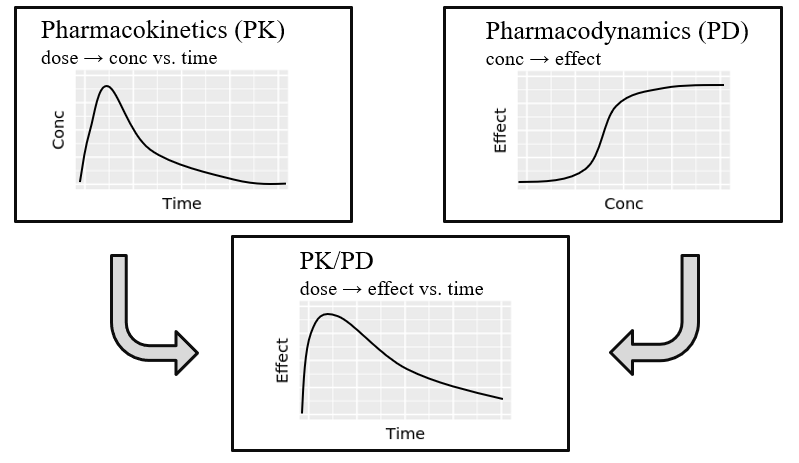

Supplement: S1 Fig — top left: Relationship between time and plasma concentration given a single dose, top right: Relationship between plasma concentration and effect given a single dose, bottom: Relationship between time and effect given a single dose (based on [38]). [file pcbi.0318549.s002.tif]

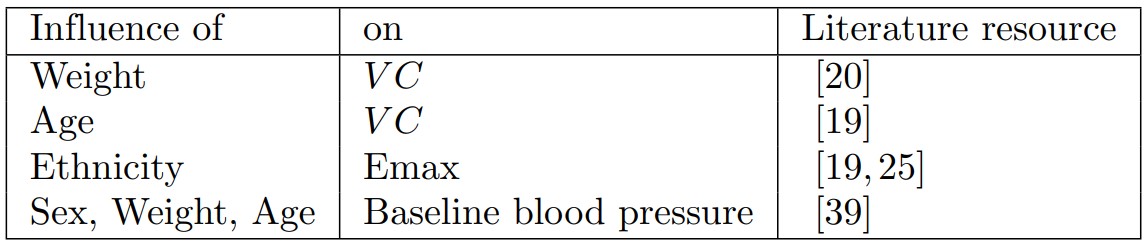

Supplement: S2 Table — Based on Fig 5. [file pcbi.0318549.s003.jpg]

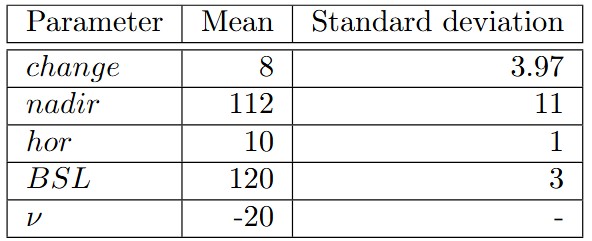

Supplement: S3 Table — The parameters for the average circadian rhythm are determined based on the parameter estimators provided by [39] and are further modified by the parameters derived from the average circadian rhythm observed in the internal study (see Fig 4). [file pcbi.0318549.s004.jpg]

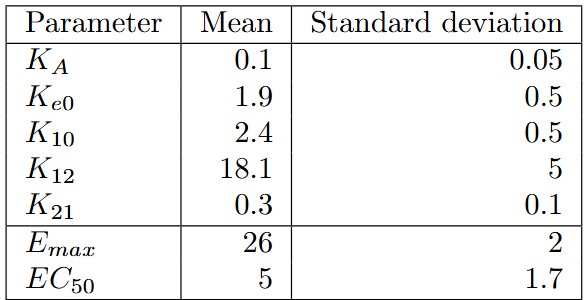

Supplement: S4 Table [file pcbi.0318549.s005.jpg]

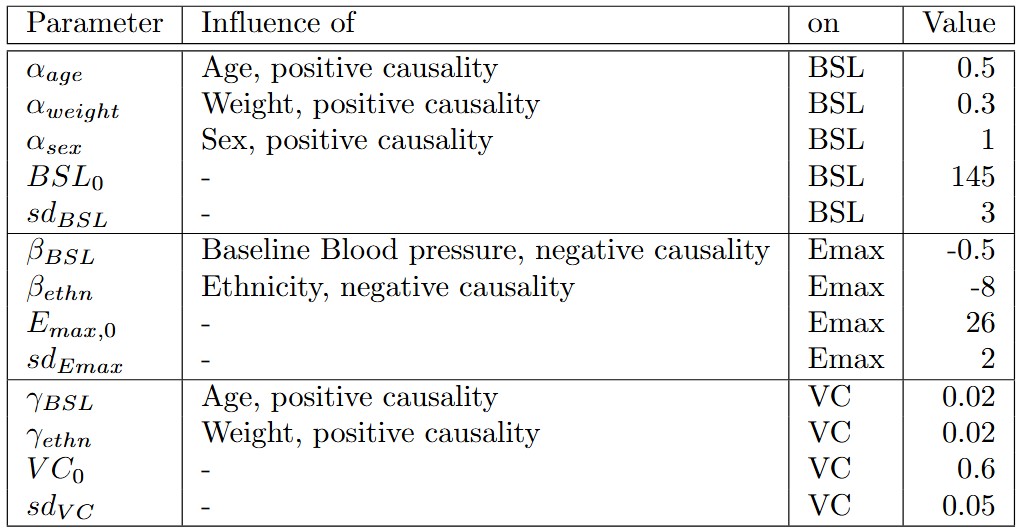

Supplement: S5 Table [file pcbi.0318549.s006.jpg]

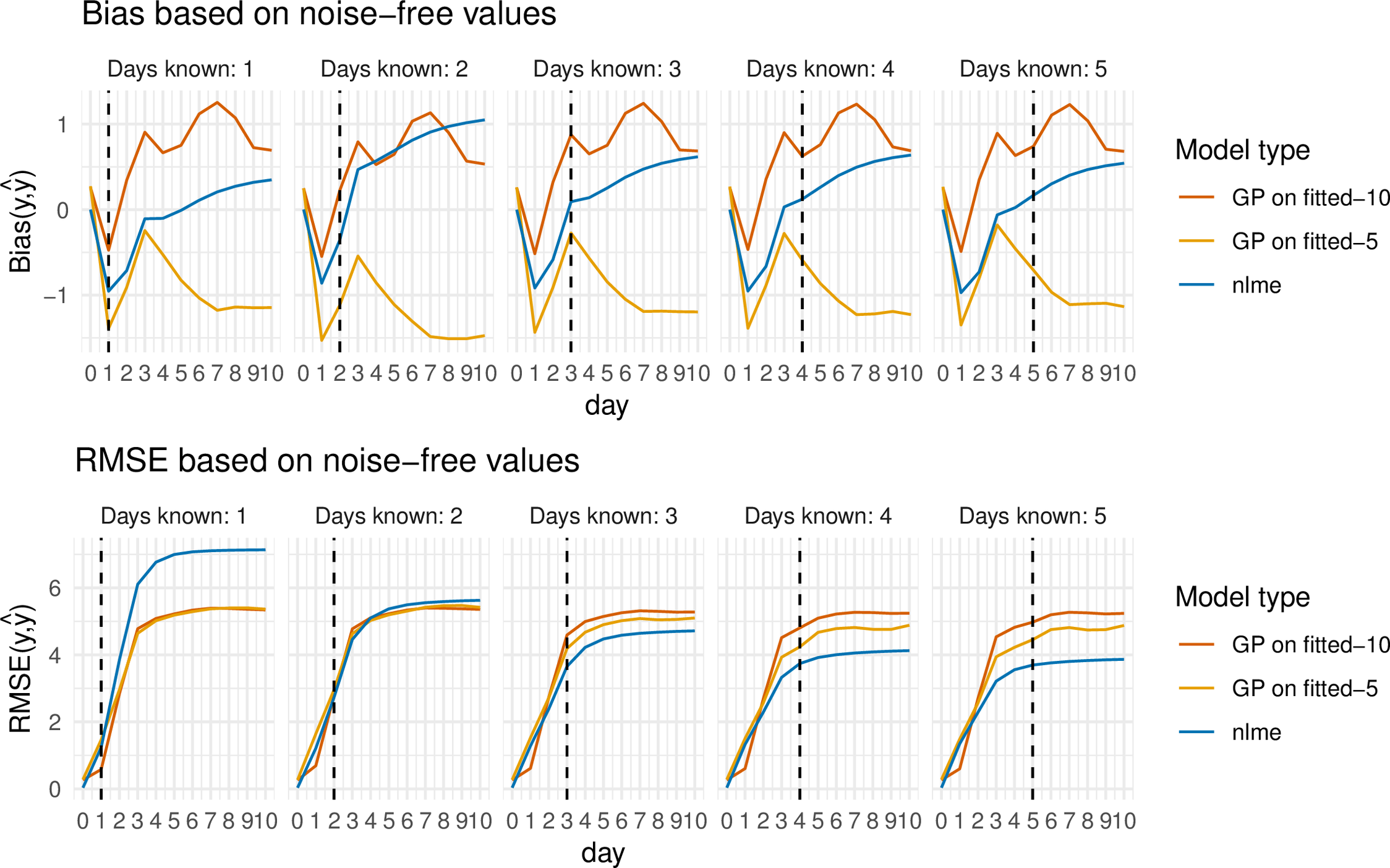

Supplement: S2 Fig — Root mean squared error (RMSE) and bias across days (t = 0… , 10 ) based on values without noise, models (non-linear mixed-effects model (nlme) in blue, advanced Gaussian process (GP) with five resp. ten nearest neighbours in yellow resp. red), and known measurements (tmax=1,…,5). [file pcbi.0318549.s007.tif]

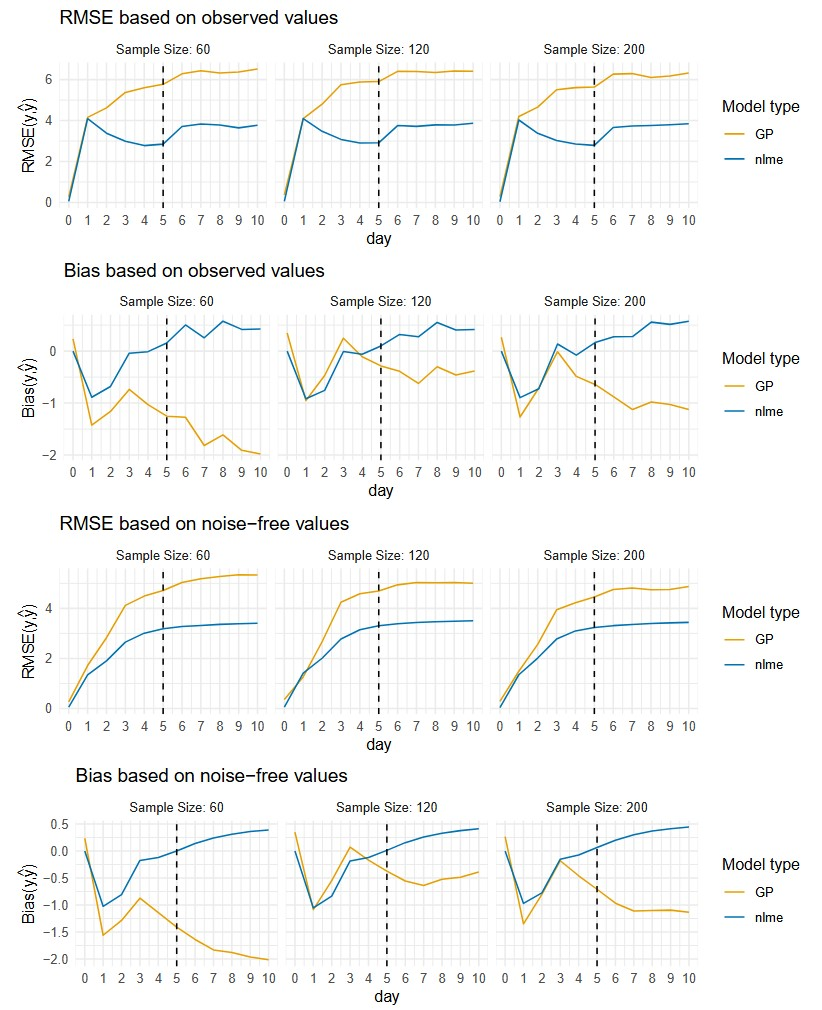

Supplement: S3 Fig — Given five days of measurements: (Non-linear in yellow) mixed-effects model (nlme) in blue, advanced Gaussian process (GP) with five nearest neighbours. We assess the impact of different sample sizes on the model performance using the five nearest neighbours for the GP model. Three different scenarios are analysed, given by sample sizes of N = 60, N = 120, and N = 200. Again, for robustness 50 datasets are simulated and assessed for each case. The RMSE as well as the bias are evaluated for noise-free and noisy values given 5 days of measurements. It can be seen that increasing the sample size will not change the models performance in terms of the RMSE, neither looking at the noisy nor the noise-free values. Looking at the bias, a higher sample size does not change the bias of the nlme model. Using the GP model, a higher sample size can reduce the bias. This suggests that, given the data assumptions (such as noise, rhythm, effect size, etc.), a sample size of approximately 120 patients would be sufficient to make an adequate prediction based on 5 days of provided measurements. [file pcbi.0318549.s008.tif]
