# Supplementary material for: A simulation-based framework for modeling and prediction of personalized blood pressure trajectories in hypertensive patients after antihypertensive treatment
Source: PLoS One. 2025 Apr 10;20(4):e0318549. doi: 10.1371/journal.pone.0318549 (PMC11984981; doi:10.1371/journal.pone.0318549)
Supplement: Attachment — Submitted filename: Response to Reviewers.pdf [file pone.0318549.s002.pdf]

Response to Reviewers

Dear reviewers,

Thank you for your helpful comments. We apologize for the errors in the manuscript. In response to your suggestions, we have revised the mentioned parts in the updated manuscript. We carefully considered the comments and tried our best to address every one of them. We hope that these changes and the enhanced text will meet your expectations. The changes have been highlighted in green (added) and yellow (changed).

Below we highlighted the aspects that have been changed in green for added aspects and in yellow for changed aspects.

Sincerely

Berit Hunsdieck

berit.hunsdieck@bayer.com

PhD Student

Reviewer #1: The manusript is written well including correct mathematical expressions. However, i have a few suggestions to make:

1. Abstract: first few sentences of abstract contain useless information which can be shifted to introduction. The abstract must revise the abstract completely by highlighting the significance of this work carried and some of key results obtained from this study.

- The abstract is revised as followed, whereas in green the added parts are highlighted and in yellow the shortened part:

  "Hypertension, a leading global cause of death, poses challenges in stabilizing blood pressure within target values despite various therapeutic options, often necessitating multiple therapy adjustments and delayed impact assessments. Recently, the first wrist-based wearable blood pressure measurement devices were introduced which allow for a continuous assessment of blood pressure trajectories. This enables the development of statistical methodology for prediction of saturated steady-state of blood pressure under treatment – and thus allowing physicians to adjust the therapy earlier. As a prerequisite for the evaluation of such models and algorithms, it is necessary to simulate reliable and realistic hypothetical patient trajectories under treatment with antihypertensive medication. In this paper, we propose a simulation framework for blood pressure profiles through Pharmacokinetic-Pharmacodynamic modeling, which incorporates individual daily rhythms, patient characteristics, and medication effects. We also propose and evaluate two models for steady-state prediction under antihypertensive therapy, a Gaussian process and a non-linear mixed effect model. When only one day of measurements is available, the Gaussian process is preferred, but in real-world situations with more data, the non-linear mixed effect model is favored. It effectively

reduces RMSE and bias in noisy data, outperforming the Gaussian process regardless of sample size."

2. Data in first 3 sentences of the introduction needs correction of data as per recent literature.

- Yes, that's right, I updated it as followed:

  "Globally, the number of hypertensive patients requiring anti-hypertensive medication increased from 594 million in 1975 to 1.3 billion in 2019, mainly in low- and middle-income countries (relative to growth in population) (WHO(2021)). Starting at a blood pressure of 115/75 mmHg, the risk of death from a heart attack or stroke at ages 35 to 69 years doubles with every 20-point increase in systolic blood pressure, making it the leading cause of death (WHO(2023))."

3. In the introduction, mention which blood pressure monitoring devices have been developed in the recent past and author must discuss the key developments in a paragraph by proper citing the literature.

- Newly added:

  "Current cuffless devices on the market are for example a chest patch developed by BioBeat Technologies (Eisenkraft et al., 2023), a wrist-wearable device developed by Aktiia (Sola et al., 2021), and a CAR-T ring developed by Skylabs (Kim et al., 2024)."

4. Mention the accuracies of wrist based blood pressure monitoring devices.

- Added:
  "Although none of these devices were accurately measuring blood pressure, they highlighted the need of re-evaluating the current practice of relying on a single blood pressure measurement in clinical settings (Schutte, 2024)."

5. Last paragraph of the introdcution is seems to be copied from a thesis or book. revise the introduction carefully by incorporating compititive studies.

- Last paragraph deleted.

6. Figure numbers are not in order. Check the citations of Fig. 2.

- Citation mistakes are corrected.

7. Figure 3 is not found in the manusript.

- Figure added to revised manuscript, citation mistakes are corrected.

8. Literature described or followed are old and outdated e.g. two compartment model outlined in literature of 2004 and 2008. The simulations are fast updating everyday. Thus authors could have folllowed recent model.

- Additional justification added:

"Although there are already complex compartment modeling approaches, a two-compartment model will be applied in the following analysis since this model is mainly used for modeling medication effects of blood pressure lowering drugs (Baek et al., 2008b; Heo et al., 2016b; Rijn-Bikker et al., 2013)."

Reviewer #2: The manuscript has some merits in terms of theoretical content whereas it seems to be poorly written in terms of language, which I feel must be addressed carefully by proofreading.
1. Abstract to be revised thoroughly for better clarity. Some key results of simulation study have to be presented here.

- Abstract is revised as followed (see Point 1, reviewer 1):
  "Hypertension, a leading global cause of death, poses challenges in stabilizing blood pressure within target values despite various therapeutic options, often necessitating multiple therapy adjustments and delayed impact assessments. Recently, the first wrist-based wearable blood pressure measurement devices were introduced which allow for a continuous assessment of blood pressure trajectories. This enables the development of statistical methodology for prediction of saturated steady-state of blood pressure under treatment – and thus allowing physicians to adjust the therapy earlier. As a prerequisite for the evaluation of such models and algorithms, it is necessary to simulate reliable and realistic hypothetical patient trajectories under treatment with antihypertensive medication. In this paper, we propose a simulation framework for blood pressure profiles through Pharmacokinetic-Pharmacodynamic modeling, which incorporates individual daily rhythms, patient characteristics, and medication effects. We also propose and evaluate two models for steady-state prediction under antihypertensive therapy, a Gaussian process and a non-linear mixed effect model. When only one day of measurements is available, the Gaussian process is preferred, but in real-world situations with more data, the non-linear mixed effect model is favored. It effectively reduces RMSE and bias in noisy data, outperforming the Gaussian process regardless of sample size."

2. Keywords are not appropriate.

- Keywords are changed to:
  Simulation-Based Trajectory Analysis, Gaussian Processes, Mixed-Effects Models, Pharmacokinetic-Pharmacodynamic Modeling, Individual Blood Pressure Monitoring

3. Introduction needs drastic revisions by incorporating discussion of key literature and models presented.
- Literature review on existing model is combined with introducing the new methods in the chapters "Simulation" and "Methods".

4. Also add a small paragraph on the key highlight of the work and novelty of the work.
- Added:

  "Based on simulated data incorporating the circadian rhythm, PKPD effects and measurements errors, the different models are evaluated. When only a single day of measurements is available, the Gaussian process is the preferred choice; however, in real-world scenarios with more data, the non-linear mixed effect model is preferred. It significantly lowers RMSE and bias in noisy data, surpassing the performance of the

Gaussian process regardless of sample size. Integrating the algorithm into wearable devices presents an innovative way to monitor medication adherence in hypertensive patients. It can predict the likelihood of achieving blood pressure reduction goals within the next day, allowing for timely adjustments to dosage and medication if necessary."

5. Correct the citation of Figure 2 in the text.

- Citation mistakes are corrected.

6. Fig. 3 is missing.

- Figure added to revised manuscript, citation mistakes are corrected.
